# Salvage surgery following immuno-chemo-radiotherapy for advanced non-small cell lung cancer

**DOI:** 10.1186/s40792-022-01371-3

**Published:** 2022-01-21

**Authors:** Ayako Nawashiro, Fumihiro Tanaka, Akihiro Taira, Shinji Shinohara, Masaru Takenaka, Koji Kuroda, Shohei Shimajiri

**Affiliations:** 1 Second Department of Surgery, University of Occupational and Environmental Health, Iseigaoka 1-1, Yahata-nishi, Kitakyushu, 807-8555 Japan; 2 Department of Pathology, School of Medicine, University of Occupational and Environmental Health, Iseigaoka 1-1, Yahata-nishi, Kitakyushu, 807-8555 Japan

**Keywords:** Salvage surgery, Lung cancer, Immunotherapy, Chemotherapy, Radiotherapy

## Abstract

**Background:**

Salvage surgery following definitive radiotherapy or systemic treatment has become a feasible treatment option in selected patients with advanced initially unresectable non-small cell lung cancer. Recent clinical trials of neoadjuvant treatment have showed that surgery following immuno-chemotherapy is safely performed. Here, we present the first case of salvage surgery following immuno-chemotherapy with concurrent definitive radiotherapy for advanced lung large cell carcinoma.

**Case presentation:**

A 44-year male was admitted to our hospital for salvage surgery. Ten months prior to this administration, he had been diagnosed with unresectable large cell carcinoma with malignant pericardial effusion (clinical stage IVA/T3N2M1A; no driver-gene alteration) originating from the right upper lobe (RUL). Due to rapid intrabronchial tumor growth causing severe dyspnea, emergency bronchial stenting in the right main bronchus using an expandable metallic stent had been performed. Thereafter, he had received immuno-chemotherapy with concurrent definitive radiotherapy. Despite dramatic radiographic response, he had suffered from persistent and refractory *Pseudomonas aeruginosa* lung infection associated with bronchial stent placement. As pericardial effusion had disappeared and no distant metastasis had developed, he was diagnosed with a potentially curable disease and was referred to our hospital. An extended sleeve resection was successfully performed, and pathological sections revealed that pathologic complete response was achieved with immuno-chemo-radiotherapy. The patient received no subsequent treatment, and is alive without tumor recurrence at 8 months after surgery.

**Conclusions:**

Salvage surgery following immuno-chemotherapy with concurrent definitive radiotherapy for advanced non-small cell lung cancer may be feasible in selected patients, and may be considered as a treatment option to control local disease.

**Supplementary Information:**

The online version contains supplementary material available at 10.1186/s40792-022-01371-3.

## Background

Salvage surgery following definitive radiotherapy or systemic treatment has been increasingly adopted as a feasible treatment option in selected patients with advanced non-small cell lung cancer (NSCLC) which had been initially diagnosed as unresectable disease [1, 2]. Recent clinical trials of neoadjuvant treatment for resectable NSCLC have revealed that the addition of immuno-therapy using an antibody against programmed death 1 (PD-1) or programmed death-ligand 1 (PD-L1) to platinum-based chemotherapy (immuno-chemotherapy) may provide superior pathologic response and superior survival benefit over chemotherapy alone [3–6]. To further improve the therapeutic efficacy, immuno-chemotherapy with concurrent radiotherapy (cICRT) may be a promising treatment option. However, the feasibility of surgery following cICRT remains unknown. Here, we present the first successful case of salvage surgery following cICRT in which pathologic complete response (pCR) was achieved with cICRT for advanced NSCLC.

## Case presentation

A 44-year male was admitted to our hospital for salvage surgery. The patient had quit smoking 4 years ago after a 20-pack-year history.

**Fig. 1:**
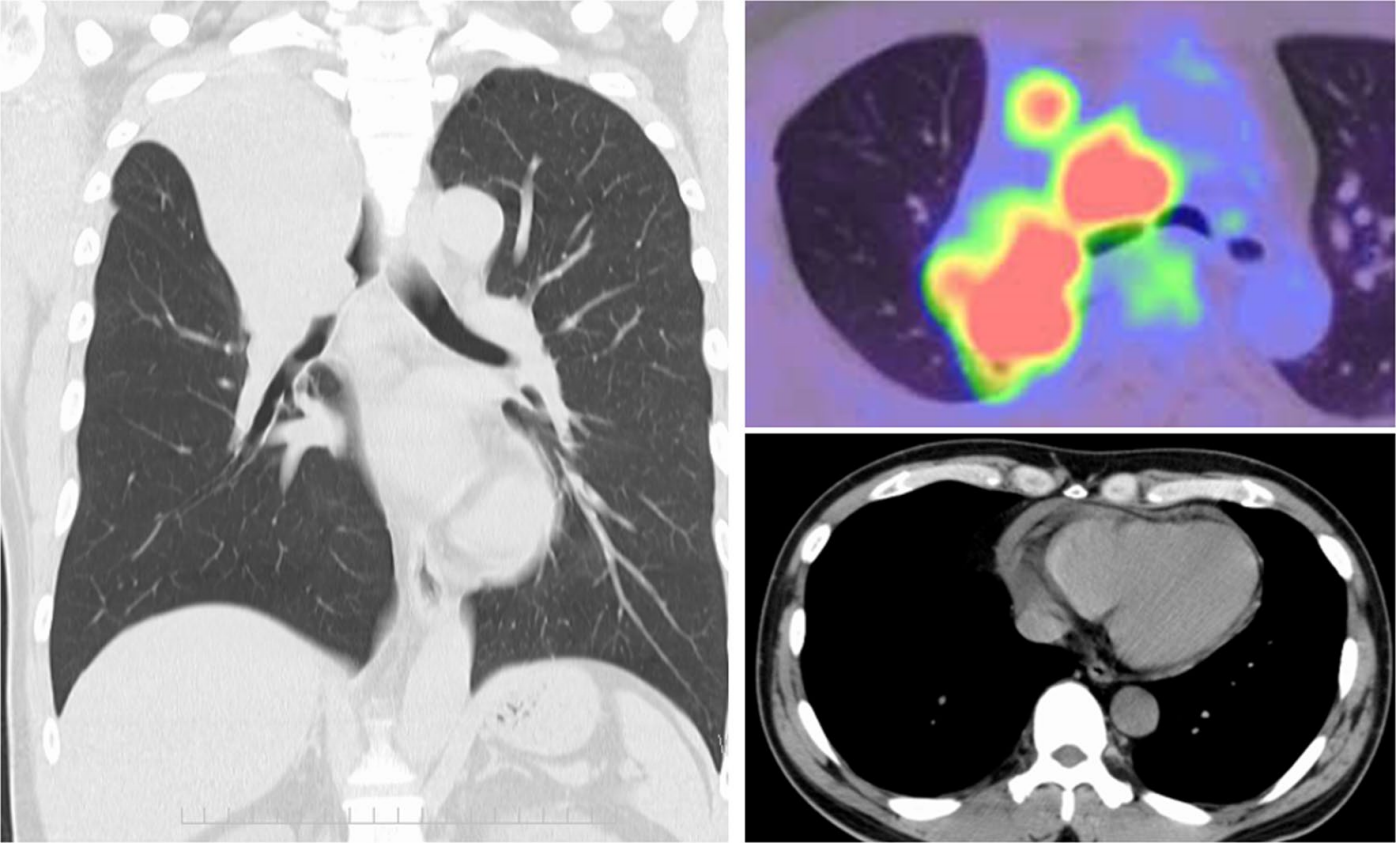
Computed tomography and positron emission tomography at initial diagnosis revealed a large tumor obstructing the right main bronchus (left), enlarged upper and lower paratracheal nodes with strong uptake of fluorodeoxyglucose (right upper), and pericardial effusion (right lower)

**Fig. 2:**
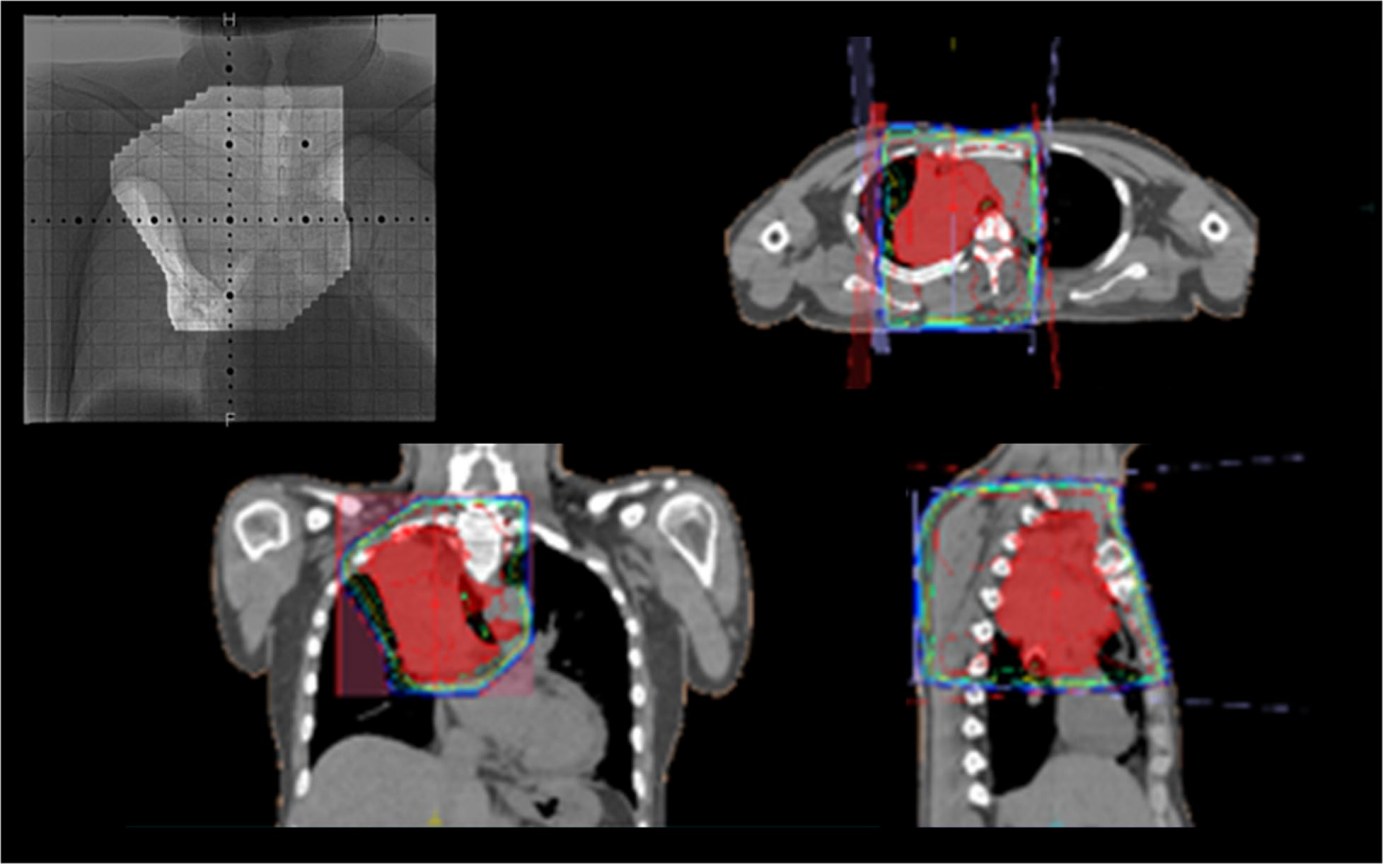
A total dose of 60 Gy in 30 fractions were delivered to the primary tumor plus involved nodes. The endobronchial stent was included in the radiotherapy field

**Fig. 3:**
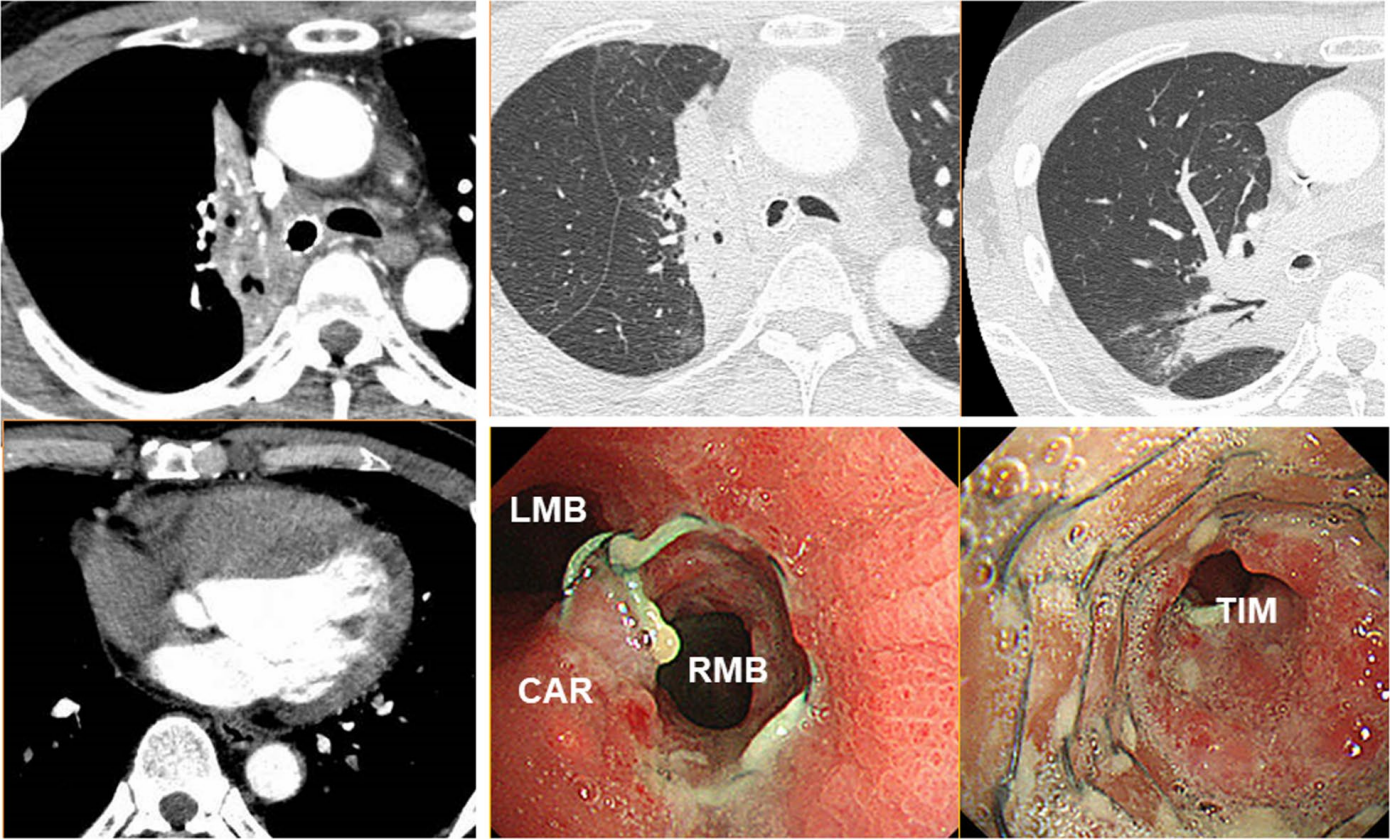
Computed tomography (CT) after bronchial stenting and immuno-chemo-radiotherapy showed dramatic tumor shrinkage and disappearance of pericardial effusion (left), and also showed persistent lung infection (right upper) associated with sent placement (right lower). Microbiological test of the “green–blue” sputum collected by deep coughing and by bronchoscopy revealed bacterial phagocytosis by neutrophils and growth of bacteria that was identified as *Pseudomonas aeruginosa*. CAR, carina; LMB, left main bronchus; RMB, right main bronchus; TIM, truncus intermedius

Ten months prior to the administration, he had been diagnosed with unresectable non-small cell lung cancer (clinical stage IVA/T3N2M1A) originating from the right upper lobe (RUL). Chest computed tomography (CT) had revealed a 4.8-cm tumor obstructing the right main bronchus, enlarged upper and lower paratracheal nodes, and pericardial effusion (Fig. 1). Transbronchial tumor biopsy had provided a pathological diagnosis of large cell carcinoma (no driver-gene alteration; proportion of tumor expressing PD-L1, 1%). Pathological evaluation of mediastinal nodes or pericardial effusion had not been performed. Due to rapid intrabronchial tumor growth causing severe dyspnea, emergency bronchial stenting in the right main bronchus using an expandable metallic stent (Ultraflex, Boston Scientific, Watertown, MA) had been performed. Thereafter, he had received immuno-chemotherapy (cisplatin plus pemetrexed with an anti-PD-1 antibody [pembrolizumab]) with concurrent definitive radiotherapy (60 Gy in 30 fractions, Fig. 2), and then had received maintenance pembrolizumab treatment. In total, he received 7 cycles of pembrolizumab treatment. Despite dramatic radiographic response, he had suffered from persistent and refractory *Pseudomonas aeruginosa* infection in the RUL and upper segment of the right lower lobe (S6) associated with bronchial stent placement (Fig. 3). As pericardial effusion had disappeared and no distant metastasis had developed, he was diagnosed with a potentially curable disease and was referred to our hospital.

Whole-body CT and fluorodeoxyglucose-positron emission tomography (FDG-PET) revealed no nodal or distant metastasis, and the patients was diagnosed to have a curative disease after cICRT (ycIB/T2aN0M0). Preoperative pulmonary function tests revealed moderate lung airflow obstruction (forced vital capacity [FVC], 3600 mL; percentage of the predicted value of FVC [%FVC], 81%; forced expiratory volume in 1 s [FEV1], 2310 mL; percentage of the predicted value of FEV1 [%FEV1], 60%; FEV1/FVC, 64%) and mild reduction of diffusing capacity of the lung for carbon monoxide (DLCO, 58% of the predicted value). The blood oxygen saturation on room air at rest was normal (97%). The patient has not had any comorbidity other than obstructive pulmonary disease. Based on these results, we decided to perform a salvage surgery to control the persistent pulmonary infection and to achieve complete resection for the cure.

**Fig. 4:**
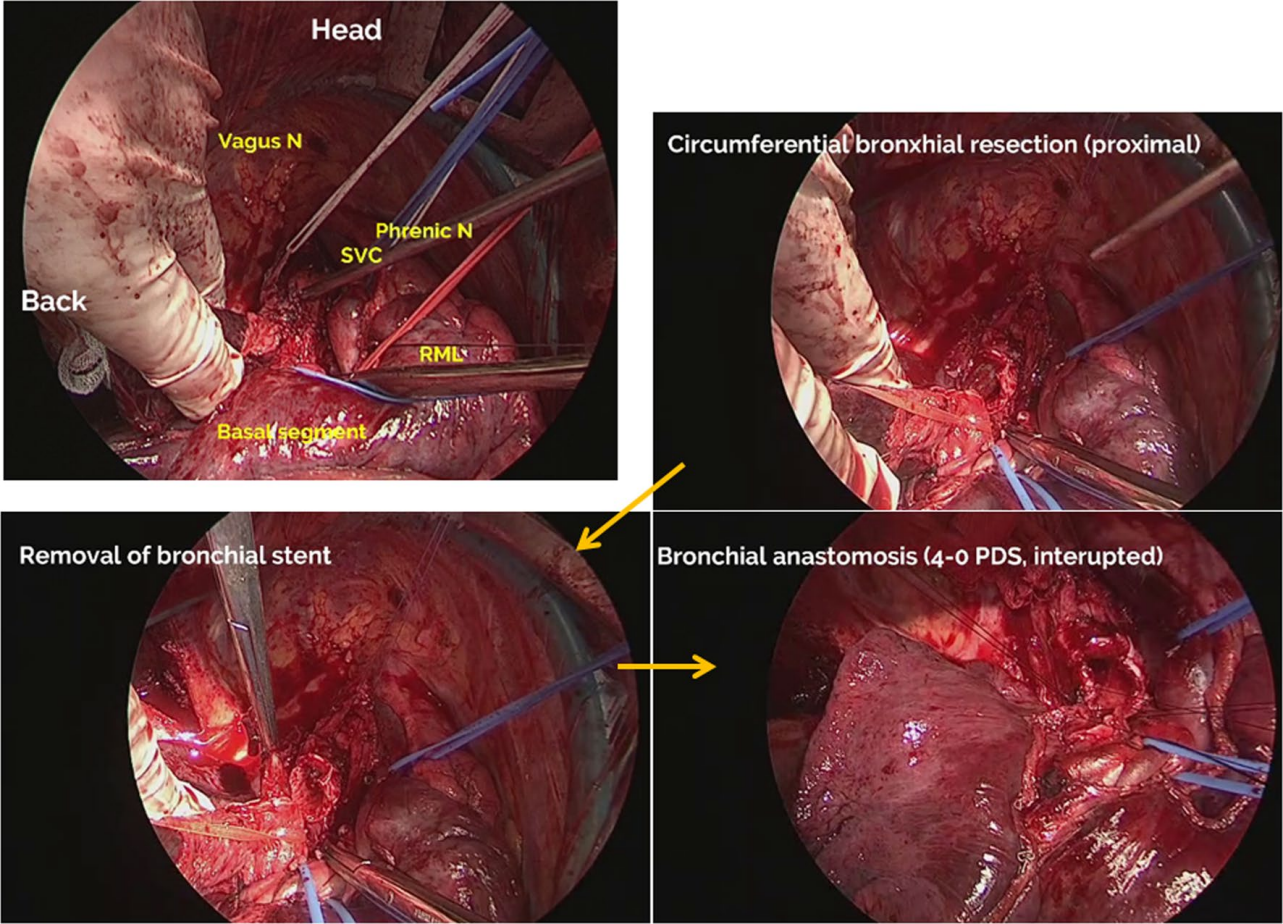
Through posterolateral thoracotomy, pulmonary vessels were divided and standard nodal dissection was completed. Then, right main bronchus and bronchus intermedius were exposed (left upper). Proximal transection of the main bronchus was performed (right upper), and the bronchial stent was removed (left lower). After distal transection of the bronchus intermedius and pathological confirmation of no malignant cells at the proximal and distal bronchial stumps, the bronchial ends were anastomosed end-to-end with interrupted 4–0 polydioxanone sutures (right lower)

Through posterolateral thoracotomy, an extended sleeve resection of RUL plus S6 was performed due to severe fibrosis and persistent infection in S6 (Fig. 4). After severe adhesion was carefully dissected, pulmonary vessels were divided and standard nodal dissection was performed. As a simple suture ligation of the first branch of the right pulmonary artery (PA), the truncus anterior (A1 + 3), was difficult, a tangential resection was performed. After clamping the right main PA, the PA segment was excised and the defect was closed by a continuous 5–0 prolene (Ethicon Inc., Somerville, NJ) suture. Circumferential bronchial resection was performed, and the stent was removed. Frozen section examination confirmed that the proximal and distal bronchial stumps were pathologically tumor free. The bronchial ends were anastomosed end-to-end with interrupted 4–0 polydioxanone (PDS, Ethicon Inc., Somerville, NJ) sutures, and were covered with a pedicled flap of pericardial fat. To relief tension of the bronchial anastomosis site, division of pulmonary ligament and pericardial incision were performed (Additional file 1).

**Fig. 5:**
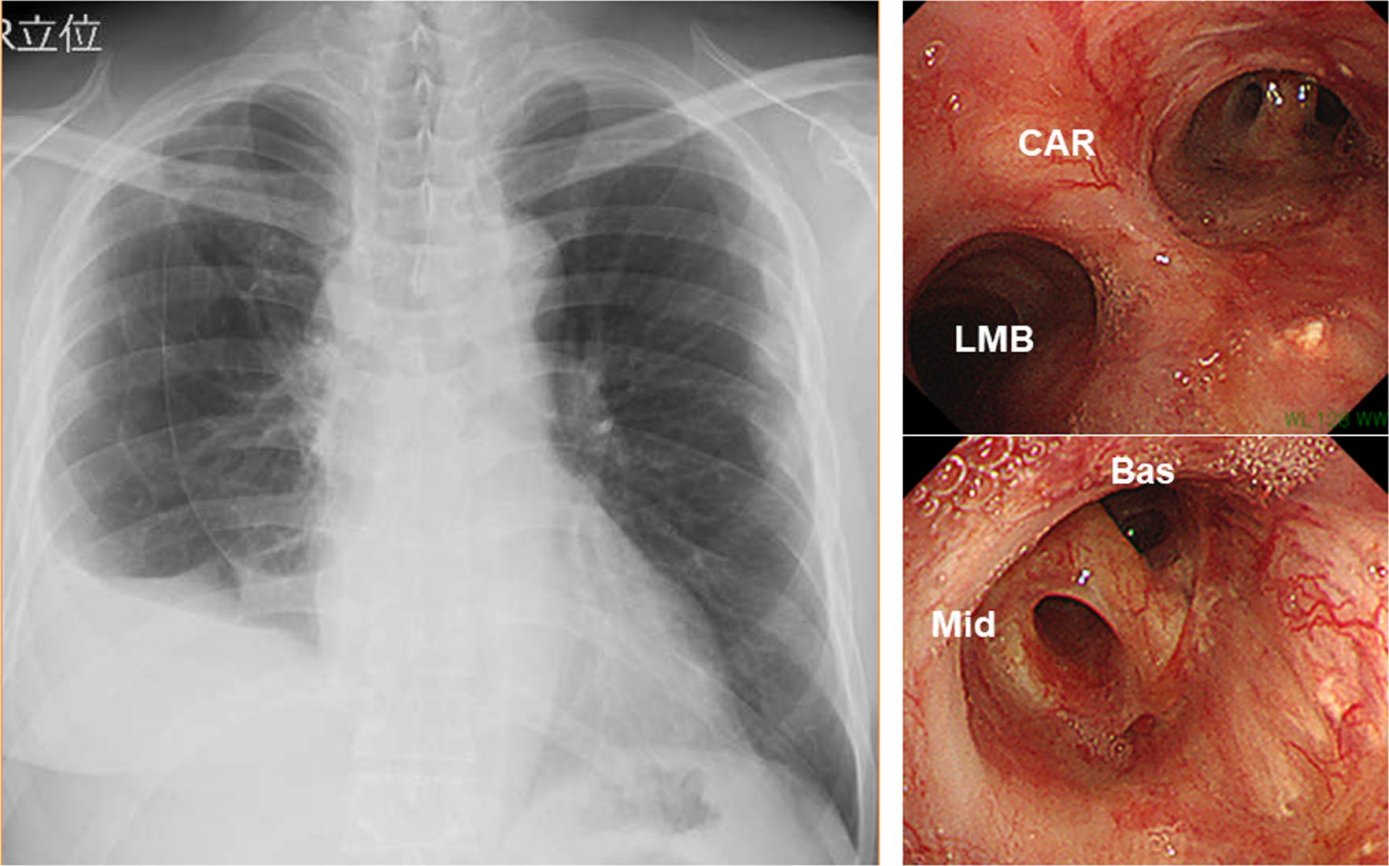
Chest roentgenogram (left) and bronchoscopy (right) at 4 months after surgery showed excellent lung expansion and bronchial healing. *CAR* carina, *LMB* left main bronchus, *Mid* right middle bronchus, *Bas* right basal bronchus

Pathological examination revealed no viable tumor cell in any resected specimen including pericardial effusion. Severe fibrosis with infiltration of a number of inflammatory cells was found in the resected lung including S6, which indicated the diagnosis of radiation pneumonitis and chronic infection. The postoperative course was uneventful, and bronchoscopy at 2 months after operation revealed excellent healing with good patency (Fig. 5). The patient received no subsequent treatment, and is alive without tumor recurrence at 9 months after surgery.

## Discussion

Immuno-chemotherapy has become a standard treatment of care for unresectable NSCLC with no driver-gene alteration [7], and also may be useful as neoadjuvant treatment prior to surgery for resectable NSCLC [3–6]. A randomized controlled trial (CheckMate 816) comparing neoadjuvant immuno-chemotherapy with chemotherapy showed that the addition of immunotherapy did not increase incidence of postoperative adverse events. The pCR rate in the immuno-chemotherapy group was significantly higher (24.0% versus 2.2%) but still remains comparable with that achieved with neoadjuvant chemo-radiotherapy [8]. In the present case, pCR was achieved with cICRT even for initially unresectable disease, which may indicate that cICRT is more effective as neoadjuvant treatment for resectable and locally advanced NSCLC. Minegishi and coworkers recently reported a successful case of salvage surgery following maintenance treatment with an anti-PD-L1 antibody (durvalumab) following concurrent chemo-radiotherapy (cCRT) for initially unresectable locally advanced NSCLC [9]. In the present case, immunotherapy was concurrently prescribed with chemotherapy in addition to definitive dose of RT, and is the first case of salvage surgery following cICRT.

In the present case, definitive RT was performed in combination with systemic treatment. However, systemic treatment without RT is generally recommended as a standard treatment for clinical stage IVA disease with pericardial effusion. The patient had complained of severe dyspnea caused by rapid intrabronchial tumor growth, and had needed rapid symptom relief with effective local treatment. Accordingly, local treatment consisting RT following bronchial stenting was performed in the present case. Systemic immuno-chemotherapy without RT might provide a favorable pathologic response. The efficacy and safety of cICRT shall be examined in future clinical trials, such as the ESPADURVA trial [10, 11].

Safety concern exists regarding surgery following cICRT despite its potential advantages of superior anti-tumor effects. The present case indicates that surgery, even extended sleeve resection with bronchoplasty, after cICRT may be safely performed. In addition, definitive-dose radiotherapy was performed concurrently with immuno-chemotherapy in the present case. Recent clinical studies have shown that surgery following chemotherapy with concurrent definitive-dose of radiotherapy is safely performed [12, 13]. Again, the safety of surgery following “definitive” cICRT shall be extensively examined in future clinical trials [10].

Airway stents may be associated with significant complications, such as persistent infection as shown in the present case, and their removal is sometimes needed [14]. Some previous studies have reported that bronchoscopic removal is safe and feasible, but is sometimes associated with fetal complications, such as severe bleeding [15, 16]. In the present case, the metallic stent was easily removed after dissecting right main bronchus through thoracotomy. We have had no experience of removing a metallic bronchial stent either through thoracotomy or under bronchoscopy, and we dissected all vessels before removing the stent to prevent severe bleeding during surgery. In addition, we had planned to perform right pneumonectomy when the stent could not be safely removed.

## Conclusions

Salvage surgery following “definitive” cICRT for advanced NSCLC may be feasible in selected patients, and may be considered as a treatment option to control local disease.

## Supplementary Information

**Additional file 1.** Salvage surgery video.

## Data Availability

All data generated during this study are included in this published article.

## Abbreviations

NSCLC: Non-small cell lung cancer
PD-1: Programmed death 1
PD-L1: Programmed death-ligand 1
cICRT: Immuno-chemotherapy with concurrent radiotherapy
pCR: Pathologic complete response
RUL: Right upper lobe
CT: Computed tomography
FDG-PET: Fluorodeoxyglucose-positron emission tomography
FVC: Forced vital capacity
FEV1: Forced expiratory volume in 1 s
PA: Pulmonary artery
